# The Diagnostic Performance of Afirma Genomic Sequencing Classifier in Black and Hispanic Patients With Indeterminate Thyroid Nodules

**DOI:** 10.1002/ohn.70400

**Published:** 2026-08-17

**Authors:** Peter J. Attia, Sree Chinta, Joseph Celidonio, John Sebastian De Armas, Evelyne Kalyoussef, Dylan F. Roden

**Affiliations:** ^1^ Department of Otolaryngology–Head and Neck Surgery Rutgers New Jersey Medical School Newark New Jersey USA

**Keywords:** indeterminate thyroid nodules, next generation sequencing, racial disparities, thyroid cancer, thyroidectomy

## Abstract

**Objective:**

About 20% of thyroid nodules detected on ultrasound are cytologically indeterminate (Bethesda categories III and IV), but the rate of malignancy of these nodules is less than 36%. The ability of Afirma Genomic Sequencing Classifier (GSC) to further risk‐stratify indeterminate thyroid nodules among Black and Hispanic patients is understudied.

**Study Design:**

Retrospective Chart Review.

**Setting:**

Two Academic Medical Centers.

**Methods:**

Black and Hispanic patients who underwent Afirma GSC from 2017 to 2024 were included. Demographics, clinical history, GSC results, and surgical pathology reports were captured. Univariate analysis was conducted to elucidate demographic and clinical characteristics of this population, and confusion matrices evaluated GSC test performance.

**Results:**

160 patients were included in this cohort with a median age at diagnosis of 51 years, 142 (88.8%) females, 86 (53.8%) Black, and 74 (46.2%) Hispanic. Median nodule size was 2.4 cm, 167 were Bethesda III, and 17 were Bethesda IV. Of the 184 Bethesda III and IV thyroid nodules tested with GSC, 131 (71.2%) were reported as benign. In total, 59 nodules (29.4%) were resected, with 19 malignancies. The observed values of sensitivity, specificity, positive predictive value (PPV), and negative predictive value (NPV) were 89.5%, 78.2%, 32.1%, and 98.5%. For all specimens with oncocytic cytology (n = 45), the sensitivity, specificity, PPV, and NPV were 83.3%, 69.2%, 29.4%, and 96.4%, respectively.

**Conclusions:**

This represents the largest study of Afirma GSC involving Black and Hispanic patients. In this cohort, the diagnostic performance of the Afirma GSC was comparable to that observed in the original validation study.

Thyroid cancer is the third fastest rising cancer diagnosis in the United States, with an estimated 3% annual increase in prevalence over the past 30 years.^1^ Thyroid nodules can be detected by ultrasound in about 50% of the general population, and the rate of malignancy confirmed by surgical pathology is estimated to be between 5% and 15%.^2^, ^3^, ^4^ The workup of thyroid nodules usually consists of obtaining thyroid‐stimulating hormone (TSH) level and an ultrasound, and performing fine needle aspiration (FNA) in accordance with the Thyroid Imaging Reporting and Data System (TIRADS) or American Thyroid Association (ATA) criteria.^5^ The Bethesda System for Reporting Thyroid Cytopathology (TBSRTC) established standardized reporting terms for FNA specimens, with about 20% of nodules being characterized as cytologically indeterminate thyroid nodules (ITN) classes III or IV.^6^ The national rate of malignancy confirmed by surgical pathology among these cytologically ITNs has been reported to be as high as 36% but varies by institution and cytopathologists’ experience based on the cytological threshold set for atypia of undetermined significance or suspicion for follicular neoplasm.^7^, ^8^

Without molecular testing of FNA samples, the rate of unnecessary thyroid surgery, defined as benign surgical pathology for ITN, has been found to be 56% for Bethesda III and 68% for Bethesda IV nodules.^9^ Despite low 30‐day complication rates (1.1% for lobectomy, 1.7% for total thyroidectomy), the number of costly complications is high due to the sheer volume of thyroid procedures, as over 150,000 surgeries are performed in the United States annually.^10^, ^11^, ^12^ Thus molecular markers help further risk‐stratify nodules for surgical excision and can reduce complications of avoidable thyroid surgery and healthcare expenditure.

The Afirma Genome Expression Classifier (GEC), used from 2011 to 2017, measures the mRNA expression of 167 gene patterns.^13^ It has been replaced by the Afirma Genome Sequencing Classifier (GSC), which uses next‐generation sequencing (NGS) to test for a larger panel of 593 gene mutations, 905 variants, and 235 fusions. The 2018 GSC validation study (n = 183) reported a sensitivity of 91%, specificity of 68%, negative predictive value (NPV) of 96%, and positive predictive value (PPV) of 47%.^14^ In further studies, GSC has improved the PPV of ITN to 47%‐85% (previously 16%‐57% with GEC) and the benign cell rate (BCR) to 54%‐76% (previously 27%‐78% with GEC), while maintaining a high sensitivity.^15^ Several studies have compared the performances of GEC and GSC at their respective institutions, but it is important to assess the real‐world performance of GSC in more recent years with the release of the second and third editions of TBSRTC in 2018 and 2023, respectively.^16^, ^17^, ^18^, ^19^, ^20^, ^21^, ^22^, ^23^

The 2018 GSC validation study and all but one of the post‐validation studies do not report the racial demographics of their study cohorts. Black and Hispanic patients experience documented disparities regarding adherence to clinical practice guidelines for thyroid nodule management as they are less likely to undergo FNA biopsies, have unequal access to care, and experience worse disease‐specific outcomes compared to White patients.^24^, ^25^, ^26^, ^27^, ^28^, ^29^, ^30^ The literature on racial genetic variation in thyroid cancer remains mixed, with some studies identifying variations in papillary thyroid cancer gene expression profiles and associations between genetic factors and radioiodine‐refractory thyroid cancer among African American patients.^31^, ^32^, ^33^, ^34^ In light of these findings, it is important to evaluate GSC performance specifically in these underrepresented groups.

This study will assess the performance of Afirma GSC on Bethesda III and IV nodules compared to definitive diagnosis with surgical pathology in Black and Hispanic patients at two academic medical centers. We hypothesize that in the absence of data on underrepresented minority patients in the Afirma GSC validation study, combined with known racial genetic differences in thyroid cancer, that GSC performance will be inferior in this cohort of patients of underrepresented minorities compared to that of other post‐validation studies. This study provides further insight into the growing literature regarding real‐world performance of molecular testing on FNA samples to risk‐stratify ITN.

## Materials and Methods

This study obtained all Afirma testing performed on FNA biopsy studies from one or more thyroid nodules between November 2017 and June 2024 that were completed at 2 medical centers in New Jersey. In an examination of underrepresented minority groups, only patients identified as Black or Hispanic were included. Patients who had Afirma GEC testing were excluded.

FNA biopsies were performed per ATA guidelines based on size, sonographic characteristics, and patient preference.^35^ Bethesda III or IV samples were reflexively shipped to Veracyte, Inc. for Afirma GSC testing. Surgical management was determined by treating clinicians using cytologic, molecular, and clinical findings. Surgical pathology diagnoses were performed by pathologists who have expertise in head and neck disease. Using the electronic medical record in a retrospective chart review, demographic data, including patient‐reported age, sex, and race, and clinical data, including family history (any relative) of thyroid cancer, nodule sonographic characteristics, cytology findings, surgical management, and histopathologic diagnosis, were collected. This study was approved by the Rutgers University Institutional Review Board with a waiver of informed consent, and data were entered into REDCap.^36^

A power calculation was performed using assumptions derived from the original 2018 Afirma GSC validation study, including an estimated thyroid malignancy prevalence of 20%.^37^ Given that we hypothesized that test performance in our predominantly underrepresented minority cohort would be diminished, we powered the study to detect whether sensitivity could be as low as 60% and specificity as low as 50%, compared to the original validation benchmarks of approximately 90% and 70%, respectively. Under these assumptions, a minimum sample size of 95 patients, including 19 malignant cases, was required to achieve 88.5% power for sensitivity analysis. Separately, the specificity analysis required a minimum sample size of 61 total cases, corresponding to approximately 49 benign cases and 12 malignant cases based on the assumed disease prevalence, to achieve 81.0% power.

We used a confusion matrix to assess the GSC classification model by comparing predicted values against actual values. To generate the confusion matrices based on observed nodules, sensitivity, specificity, PPV, negative predictive value (NPV), and accuracy were calculated for all nodules with exclusion of nodules that had a “suspicious” finding on Afirma GSC but no surgery. As previously reported, conservative values for specificity, PPV, and accuracy were calculated by assuming that GSC‐suspicious (GSC‐S) nodules would have been benign on surgical pathology and thus characterized as “false positives.”^38^ Subgroup confusion matrices were generated for nodules with Bethesda III cytology and the presence of oncocytic cells on FNA samples, as well as for those with no mutations reported on the Afirma Xpression Atlas (XA). A confusion matrix was generated for those nodules on which Afirma GSC was performed, but for which there was no reported gene mutation. Exact 95% confidence intervals were calculated for each of these measures using the Clopper‐Pearson method.^39^ The benign call rate (BCR) was calculated as the proportion of nodules that were benign on GSC (GSC‐B) out of the total number of nodules assessed with GSC. All analyses were performed using SPSS Software version 29.0 (IBM Corp.).

## Results

Afirma GSC was performed on 242 thyroid nodule samples from 216 unique patients between November 2017 and June 2024. 58 total nodules from 56 patients were excluded because of insufficient material for testing (20 nodules), “No Result” on GSC (8 nodules), or patient race other than Black or Hispanic (30 nodules: 12 White, 8 Asian, 10 other race) (Figure 1). Of the 184 nodules from 160 patients included, the median age at diagnosis was 51 years. There were 142 (88.8%) females, 86 (53.8%) Black, and 74 (46.2%) Hispanic patients. Relevant medical history revealed that 26 (16.3%) had a family history of thyroid cancer. The median size of the thyroid nodules assessed with GSC was 2.4 centimeters (Table 1). By Bethesda classification, 167 nodules (90.8%) were Bethesda III, and 17 (9.2%) were Bethesda IV. Following GSC testing, 131 (71.2%) were GSC‐B and 53 (28.8%) were GSC‐S (Figure 1).

**Figure 1:**
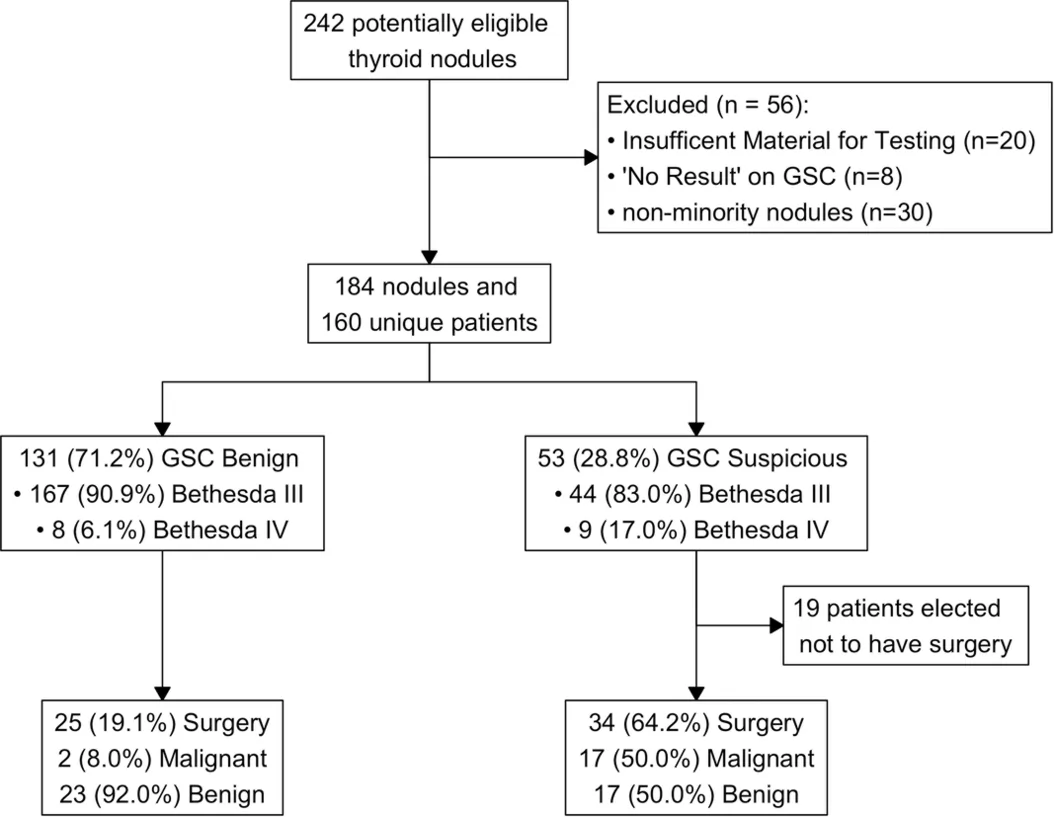
Nodule inclusion/exclusion criteria. This image depicts the patient and nodule inclusion criteria used in this study.

**Table 1:** Patient and Nodule Characteristics

Patient characteristics	
Total patients [N]	160
Age at diagnosis of patients [median (IQR), years]	51 (42‐60)
Sex [N (%)]	
Male	18 (11.2%)
Female	142 (88.8%)
Race [N (%)]	
Black	86 (53.8%)
Hispanic	74 (46.2%)
Family history of thyroid cancer, [N (%)]	26 (16.3%)
Patients who underwent surgery [N (%)]	47 (29.4%)

**Nodule characteristics**	
Total nodules [N]	184
Nodule size on US [median (IQR), cm]	2.4 (1.8‐3.5)
Bethesda category [N (%)]	
Bethesda III	167 (90.8%)
Bethesda IV	17 (9.2%)

Assuming that all nonoperative GSC‐S nodules would have been benign on surgical pathology, the diagnostic performance characteristics of Afirma GSC are as follows: sensitivity of 89.5%, specificity of 78.2%, PPV of 32.1%, NPV of 98.5%, and accuracy of 79.3% (Table 2). The diagnostic performance characteristics of GSC were similar across nodules with available surgical pathology (Table 3). The BCR of Bethesda III nodules without oncocytic cytology was 75.4% and the BCR of Bethesda III nodules with oncocytic cell nodules was 66.7% (Table 4).

**Table 2:** Confusion Matrix Comparing Malignant Versus Benign Findings on Surgical Pathology to Afirma GSC for All Thyroid Nodules (Conservative Values; Inclusion of Suspicious Nodules That Were Not Operated on and Assumed to be Benign) [n = 184]

	Malignant on pathology	Benign on pathology	
Suspicious on GSC	17 (9.2%)	36 (19.6%)	PPV = 32.1% (19.5%‐44.6%)
Benign on GSC	2 (1.1%)	129 (70.1%)	NPV = 98.5% (96.4%‐100.0%)
	Sensitivity = 89.5% (75.7%‐99.9%)	Specificity = 78.2% (71.9%‐84.5%)	Accuracy = 79.3% (72.7%‐84.49%)

Abbreviations: GSC, Genomic Sequence Classifier; NPV, negative predictive value; PPV, positive predictive value.

**Table 3:** Confusion Matrix Comparing Malignant Versus Benign Findings on Surgical Pathology to Afirma GSC for Observed Thyroid Nodules (Exclusion of Suspicious Nodules With no Surgical Pathology Confirmation, as is Usually Reported by Postvalidation Studies) [n = 165]

	Malignant on pathology	Benign on pathology	
Suspicious on GSC	17 (10.3%)	17 (10.3%)	PPV = 50.0% (33.2%‐66.8%)
Benign on GSC	2 (1.2%)	129 (78.1%)	NPV = 98.5% (96.4%‐100.0%)
	Sensitivity = 89.5% (75.7%‐99.9%)	Specificity = 88.4% (83.2%‐93.6%)	Accuracy = 88.5% (82.6%‐92.9%)

Abbreviations: GSC, Genomic Sequence Classifier; NPV, negative predictive value; PPV, positive predictive value.

**Table 4:** Confusion Matrix Comparing Malignant Versus Benign Findings on Surgical Pathology to Afirma GSC for Observed Thyroid Nodules by Hürthle Cell and Bethesda III Cytology Subgroups

Test	Sensitivity	Specificity	NPV	PPV	n	TP	FP	TN	FN
Bethesda III and IV HC+	83.3% (53.5%‐100.0%)	69.2% (54.8%‐83.7%)	96.4% (89.6%‐100.0%)	29.4% (7.8%‐51.1%)	45	5	12	27	1
Bethesda III HC−	90.0% (58.7%‐99.8%)	80.7% (73.4%‐87.6%)	99.0% (94.8%‐100.0%)	27.7% (12.1%‐42.5%)	134	9	24	100	1
Bethesda III HC+	80.0% (44.9%‐100.0%)	75.0% (59.0%‐91.0%)	95.5% (86.8%‐100.0%)	36.4% (7.9%‐64.8%)	33	4	7	21	1

Abbreviations: FN, false negative; FP, false positive; GSC, Genomic Sequence Classifier; HC, Hürthle cell; NPV, negative predictive value; PPV, positive predictive value; TN, true negative; TP, true positive.

Afirma XA detected mutations or fusions in 15 (8.0%) nodules (Table 5). All medullary thyroid carcinomas that were confirmed by surgical pathology tested positive for the MTC RNA‐sequence classifier (Table 5). The only 2 nodules with BRAF V600E mutations and surgical pathology reports available were both confirmed to be papillary thyroid carcinomas (Table 5). Excluding patients with identifiable gene mutations did not change the performance characteristics of Afirma GSC (Table 6).

**Table 5:** Clinical and Surgical Pathology Characteristics for Patients Who Had Identified Gene Mutations on Afirma Xpression Atlas

Age (years)	Sex	Race	Afirma GSC	Afirma malignancy risk percentage	Xpression Atlas Mutation	Xpression Atlas Fusion/ Variant	Surgical pathology	Surgical management
36	Female	Hispanic	Suspicious	>95%	BRAF V600E	BRAF:p:V600E c.1799T>A	No surgery	No surgery
67	Female	Hispanic	Suspicious	>95%	BRAF V600E	BRAF p. V600E c. 1799T>A	Papillary carcinoma, classic	Completion thyroidectomy
57	Female	Hispanic	Suspicious	>99%	MTC	N/A	Medullary carcinoma	Total thyroidectomy
53	Female	Hispanic	Suspicious	>95%	BRAF V600E	N/A	Papillary carcinoma, classic	Total thyroidectomy
46	Female	Hispanic	Suspicious	>99%	MTC	RET:pM918T c.2753T>C RET:pV804M c2410G>A	Medullary carcinoma	Total thyroidectomy
36	Female	Hispanic	Suspicious	>95%	BRAF V600E	BRAF p. V600E c. 1799T>A	Papillary carcinoma, classic	Total thyroidectomy
50	Female	Black	Suspicious	>99%	MTC	N/A	Medullary carcinoma	Total thyroidectomy
57	Female	Hispanic	Suspicious	50%	N/A	KRAS p.Q61R c.182A>G	Follicular carcinoma, minimally invasive	Total thyroidectomy
			Suspicious	50%	N/A	NRAS p.Q61R c.182A>G	Follicular carcinoma, minimally invasive	Total thyroidectomy
63	Male	Black	Suspicious	75%	N/A	NRAS:p.O61K c. 181C>A	No surgery	No surgery
41	Female	Black	Suspicious	75%	N/A	NRAS:p.Q61R c.182A>G	No surgery	No surgery
35	Female	Hispanic	Suspicious	75%	N/A	NRAS:p.Q61R c.182A>G	Oncocytic follicular tumor of uncertain malignant potential	Total thyroidectomy
58	Female	Hispanic	Suspicious	75%	N/A	PAX8/PPARG	Oncocytic follicular thyroid nodule of uncertain malignant potential	Left hemithyroidectomy
66	Female	Black	Suspicious	25%	N/A	TSHR:p.M453T c.1358T>C	Adenomatoid nodule	Left hemithyroidectomy

Abbreviation: GSC, Genomic Sequence Classifier.

**Table 6:** Confusion Matrix Comparing Malignant Versus Benign Findings on Surgical Pathology to Afirma GSC for Nodules With no Reported Gene Mutation in Black or Hispanic Patients (Observed)

	Malignant on pathology	Benign on pathology	
Suspicious on GSC	10 (6.4%)	15 (9.6%)	PPV = 40.0% (20.8%‐59.2%)
Benign on GSC	2 (1.3%)	129 (82.7%)	NPV = 98.5% (96.4%‐99.9%)
	Sensitivity = 83.3% (62.3%‐99.9%)	Specificity = 89.6% (84.6%‐94.6%)	Accuracy = 89.1% (83.1%‐93.5%)

Abbreviations: GSC, Genomic Sequence Classifier; NPV, negative predictive value; PPV, positive predictive value.

The surgery rate of the GSC‐B nodules was 19.1%, while the surgery rate of the GSC‐S nodules was 64.2% (Figure 1). Among 59 resected nodules, 19 (32.2%) were malignant, with common findings being classic papillary carcinoma [6 (31.6%)], encapsulated follicular variant papillary carcinoma with tumor capsular invasion [4 (21.1%)], medullary carcinoma [4 (21.1%)], and minimally invasive follicular carcinoma [3 (15.8%)].

## Discussion

The increase in knowledge of differentiated thyroid cancer driver mutations and accessibility of genetic sequencing in recent years has modified the management algorithm of thyroid nodules to include DNA‐ and RNA‐based testing.^40^ The benefit of these molecular tests, including Afirma GEC and GSC, ThyroSeq, and ThyGeNEXT/ThyraMIR, and others, is a decrease in unnecessary surgery for ITN by ruling out malignancy because of their high sensitivities and NPV.^41^ It is important to assess the real‐world performance of GSC because of the purported increase in PPV and BCR compared to GEC due to inclusion of NGS that tests for additional point and fusion mutations as well as presence of oncocytic cells.^14^, ^42^ The performance characteristics of GSC may vary by institution because of different prevalences of malignancies, nodules with oncocytic cytology, tendency for surveillance versus resection for GSC‐S nodules, and TBSRTC reporting by cytopathologists.^38^ This study aimed to report the GSC test performance among Black and Hispanic patients.

Contrary to our original hypothesis, Afirma GSC performance did not appear to be diminished in this minority cohort. One possible explanation is that the mutational landscape and expression of the 1115 core genes used in GSC's core ensemble model and for classifying a nodule as suspicious versus benign may not differ across racial/ethnic groups in ways that meaningfully affect classifier performance.^14^ This aligns with emerging paradigms suggesting that socially defined racial categories may not reliably capture underlying human genomic variation.^43^ Additionally, the broader sequencing approach used in GSC compared to earlier platforms, such as Afirma GEC, may improve robustness across diverse patient populations. Clinically, these findings suggest GSC can be applied across diverse patient populations with similar confidence without evidence of performance degradation. However, continued multi‐institutional studies with larger, more geographically diverse cohorts remain necessary to validate and confirm these findings and to evaluate subgroup‐specific differences not detectable in the current study.

It has been found that non‐White patients are more likely to have larger tumor sizes at presentation than White patients.^29^ Prior studies have demonstrated racial differences in thyroid cancer presentation and histologic subtype distribution. Black patients have been reported to have lower incidence rates of papillary, follicular, and medullary thyroid cancers compared to White patients.^44^ Therefore, it is particularly important to assess the diagnostic performance of genetic testing in ITN in Black and Hispanic cohorts because of the disparities related to thyroid nodule management, which are associated with surgical undertreatment, delayed time to referral, and lower disease‐specific survival of these groups.^45^, ^46^, ^47^

Kerr et al. also examines Afirma GSC in an underrepresented minority population and found that Afirma GSC had a PPV of 35.7% and NPV of 96.6% on 116 ITN in a study population of 56% Hispanic patients.^48^ These PPV and NPV values are similar to those of our study, as are the sensitivity of 84.6%, specificity of 81.4%, and BCR of 74%, though these three values include the performance of 11 GEC results.^48^ They also found that GSC performance in Hispanic patients was similar to that in White patients, consistent with our findings. However, Kerr et al. studied only 12 Black patients (n = 12, 8.3%), whereas our study extends these findings by evaluating GSC performance in a larger cohort of Black patients (n = 86, 53.8%). Beyond Kerr et al. only 1 other study reports Afirma GSC results by race and has a cohort of 89.5% White patients.^15^ Most other GSC studies have similar age and gender demographics, with their cohorts consisting of over 70% female patients, a median age in the mid‐50s, and nodules with a median size of 2 to 3 cm.^16^, ^17^, ^19^, ^21^, ^22^, ^49^, ^50^ It is well‐established that there is a strong correlation between both female gender and increasing age and the development of thyroid nodules, consistent with the observed middle‐aged female predominance in our study cohort.^51^, ^52^

The most recent meta‐analysis by Nasr et al. of 13 independent real‐world postvalidation studies assessing Afirma GSC diagnostic performance reveals similar performance characteristics between the aggregate of those studies and ours.^38^ This meta‐analysis did not include information about patient race or ethnicity. Our performance metrics when unoperated GSC‐S nodules were excluded at our institution (Table 5) were not significantly different from the 97.2% sensitivity, 99.5% NPV, 64.9% PPV, 87.7% specificity, and 66.9% BCR calculated in the meta‐analysis. Several studies have corroborated that the true incidence of thyroid malignancy may be lower in Black patients compared to White patients, independent of differences in socioeconomic status or frequency of FNA for incidental thyroid nodules.^30^, ^53^ Thus, the diagnostic performance characteristics that are impacted by prevalence, PPV and NPV, in this underrepresented minority cohort maintain their robustness despite a lower true incidence of thyroid malignancy in our study cohort. Indeed, the rate of malignancy of our cohort was 10.3% while the rates in most other studies was higher, ranging from 12.8% to 24.0%.^15^, ^17^, ^19^, ^21^, ^49^, ^54^ However, as is the case with the original 2018 validation study, all but two of these post‐validation studies do not report the racial demographics of their study cohorts so comparisons of the racial demographics of other studies with those of our own cannot be definitively made.^14^ Notably, our conservative PPV was lower than that which was calculated in the meta‐analysis. The rate of operation of GSC‐S nodules in our study was lower at 64.2% compared to the 78.0% reported in the meta‐analysis, possibly due to the expanding role of active surveillance in small (<1.5 cm) thyroid cancers (or suspicious thyroid cancers). Patients of different ethnic backgrounds may also have differences in cultural opinions about the need to undergo surgery for a “suspicious” diagnosis. Furthermore, this study includes six surgeons from both general surgery and otolaryngology departments, with involvement of community and academic endocrinologists with different referral patterns and shared decision‐making strategies, which may contribute to a lower resection rate.

The presence of oncocytic cells on cytological analysis has reduced the diagnostic accuracy of GEC because oncocytic cells can be present in benign thyroid disease, such as Graves' and Hashimoto's, as well as in oncocytic cell neoplasms.^55^ GSC aims to address GEC's shortcomings in the diagnostic performance of nodules with oncocytic cytology by incorporating genetic markers specific to oncocytic cells. It is important to assess the performance characteristics of GSC in our study cohort because of the higher incidence of Graves' disease in the Black population.^56^ We found that the performance of GSC for nodules with oncocytic cytology was similar to the performance of GSC for all nodules included in our study cohort, which is consistent with the oncocytic cell GSC analysis in the literature, though Livhits et al. noted that the oncocytic cell cohort in their study had a lower PPV of 20.0%.^16^, ^21^, ^49^ The performance of GSC on nodules with oncocytic cytology in this study supports that GSC has decreased the rates of false positives previously seen in GEC of these types of nodules.

Although there is a lack of data comparing the detection of genomic variants and fusions by Afirma XA to ultimate surgical pathology findings, Afirma XA has been shown to have high positive predictive agreement with targeted DNA and RNA sequencing.^57^ The Afirma MTC RNA‐sequencing classifier has been found to have 100% sensitivity and specificity for detecting medullary thyroid carcinomas, consistent with all four of the medullary thyroid carcinomas confirmed by surgical pathology in our study cohort testing positive for the MTC classifier.^58^ The ability of Afirma GSC to accurately detect MTC is important because these cancers have worse prognosis than differentiated thyroid cancer due to their aggressive growth pattern and unresponsiveness to radioactive iodine, so timely total thyroidectomy can have significant survival benefit for these patients.^59^

The demographics of the patients in the Afirma GSC validation study and almost all of the postvalidation studies are not available. Thus, it is important to verify across all ethnic groups that the rate of malignancy among GSC‐B nodules is ≤4% and that the rate of malignancy among GSC‐S nodules with no genetic variants or fusions is approximately 50%.^60^ In our study cohort of Black and Hispanic patients, the rate of malignancy among GSC‐B nodules was 1.55% and the rate of malignancy among GSC‐S nodules with no reported mutations on XA was 40.0% (95% CI: 21.1%‐61.3%), both of which are consistent with the values reported by Veracyte. Despite a lower rate of malignancy in this cohort of Black and Hispanic patients, the PPV of Afirma GSC was not adversely affected. The performance characteristics of Afirma GSC in the subcohorts of nodules both with and without reported XA mutations supports that the real‐world performance of Afirma GSC may be similar among patients of both underrepresented minority and non‐minority groups.

## Limitations

Inherent to guideline‐based management of thyroid nodules, our study lacks surgical pathology results for many GSC‐B nodules, as not all patients underwent surgical resection. Pathologists at our institution were not blinded to GSC results, which has the potential to bias the surgical pathologic diagnoses. While our oncocytic findings were comparable to the overall cohort, the sample size was underpowered, and larger studies are needed to support these findings. Due to shared decision‐making (active surveillance), patient refusal, or loss of follow‐up, not all GSC‐S nodules were resected, potentially impacting our calculated diagnostic performance characteristics. Our institution's lower rate of surgery is notable when comparing the performance of GSC in our institution to that of other institutions. This retrospective study was limited by the demographic data available within the institutional electronic medical record, which may not have fully captured multiracial or Afro‐Hispanic identities. Additionally, the institutional patient population limited subgroup analyses of other racial and ethnic groups. Although the study met prespecified power assumptions for the primary hypothesis tested, the modest sample size may have limited the detection of smaller subgroup‐specific differences.

## Conclusion

This is the largest study of the diagnostic performance of Afirma GSC in a cohort of Black and Hispanic patients. The observed sensitivity, specificity, PPV, and NPV in this underrepresented minority cohort are similar to those of Afirma GSC at other institutions, though the BCR and specificity were higher than in the validation study. The diagnostic accuracy of Afirma GSC and the high BCR (71.2%) have allowed nearly 68% of the entire cohort and 80% of patients with GSC‐B results to avoid surgery.

## Author Contributions

**Sree Chinta**, BS, data curation, formal analysis, investigation, visualization, writing (original draft), writing—review and editing; **Peter J. Attia**, BA, data curation, validation, formal analysis, visualization, writing—reviewing and editing; **Joseph Celidonio, MD**, data curation, investigation, visualization, writing—original draft, writing—review and editing; **John Sebastian De Armas, MD,** data curation, investigation, visualization, writing—original draft, writing—review and editing; **Evelyne Kalyoussef**, MD, conceptualization, methodology, supervision, writing—review and editing; **Dylan F. Roden**, MD, MPH, conceptualization, methodology, supervision, writing—review and editing.

## Disclosures

### Competing interests

None.

### Funding source

None.
